# Auricular Protrusion After the Postauricular Approach: A Review of the Current Literature

**DOI:** 10.7759/cureus.46509

**Published:** 2023-10-05

**Authors:** Mohammad Mokhatrish

**Affiliations:** 1 Depratment of Surgery, Prince Sattam bin Abdulaziz University, Al-Kharj, SAU; 2 Department of Otorhinolaryngology and Head and Neck Surgery, Prince Sattam bin Abdulaziz University, Al-Kharj, SAU

**Keywords:** dressing, mastoidectomy, ear surgery, auricular protrusion, postauricular approach

## Abstract

The postauricular approach is a widely adopted surgical technique for the ear due to its unique access to the middle ear, mastoid, and other internal structures, while adeptly concealing the surgical incision for aesthetic superiority. Despite its advantages, concerns have emerged regarding the potential for auricular protrusion following the procedure. While the exact mechanisms underlying this phenomenon remain under debate, it is worth noting that comprehensive literature on this topic is scant. Nevertheless, available studies predominantly indicate no association between the postauricular approach and lasting auricular protrusion. In the few reports that do note its occurrence, the protrusion appears transient, resolving over time. These findings suggest that surgeons should continue using the postauricular approach without concerns regarding auricular protrusion. Nonetheless, it is recommended to take all precautionary measures, including appropriate patient selection, engagement of an experienced surgeon, and meticulous postoperative dressing.

## Introduction and background

Various surgical interventions for the ear, including the transcanal, endaural, and postauricular approaches, are chosen based on the surgeon's discretion. Among these, the postauricular method is frequently employed owing to its distinct advantages [1]. This approach offers unique access to the middle ear, mastoid, and other internal structures while minimizing visible external scarring [2]. Furthermore, since the incision is placed behind the ear, it offers an aesthetic benefit by concealing the surgical incision [3]. Since its first introduction, this technique has undergone numerous modifications to enhance its efficacy and minimize complications, each iteration informed by decades of surgical experience and anatomical studies [4]. However, an underlying concern exists regarding the potential alteration of the auricular shape postsurgery due to this incision. While the auricular cartilage lies adjacent to the cranium, they are not conjoined directly. The ear's stability is maintained through a combination of skin, connective tissues, soft tissues, and the auricle's external muscles [5].

As with any surgical intervention, the postoperative outcomes, both functional and aesthetic, are of significant importance. Among these, the positioning of the auricle after surgery is particularly crucial [6,7]. A well-positioned auricle not only contributes to the overall aesthetic outcome but also plays a pivotal role in auditory function and patient satisfaction [8]. Misalignment or protrusion can result in both functional impairment and significant patient distress, emphasizing the need for precision and expertise in both the surgical technique and postoperative care. In this review, we aimed to summarize the current evidence regarding the potential implications of the postauricular approach on auricula protrusion.

## Review

Ear anatomy

Understanding the details of the auricular anatomy is pivotal for assessing auricular protrusion. Proficient knowledge of the ear's structure enables surgeons to reduce the risk of auricular protrusion after the postauricular approach and enables them to achieve optimal aesthetic outcomes.

Though there is a spectrum of anatomical variations in ear structure from one individual to another, some standard components delineate its basic framework. Predominantly, the ear consists of elements like the helix, antihelix, concha, tragus, and lobule. Additional features encompass the antitragus, intertragal incisures, and Darwin's tubercle [9]. For the typical adult, the ear spans a height of around 6 cm, with its width being roughly 55% of its height [10]. Notably, the conchal bowl is characterized by a depth of about 1.5 cm, encircled by a pronounced outer edge [10]. When observed from the front, the helix extends about 2 to 5 mm beyond the antihelix [9]. A lack of proper curvature in the antihelix can cause the helical rim to project forward, giving the ear its protruded appearance. A key indicator here is the conchoscaphal angle; if it surpasses the typical 90-degree limit due to an imperfectly folded antihelix, it can lead to enhanced ear projection [11]. As a standard, the distance of the auricle from the mastoid process shouldn't exceed 2 cm, resulting in an auriculocephalic angle of under 25 degrees. Specific measurements between the helix and mastoid range from 10 to 12 mm at the ear's upper third, 16 to 18 mm at its middle, and 20 to 22 mm at the lower third [12]. The difference in distance between the helix and mastoid for both ears is generally confined to 3 mm [12].

The ear's placement on the head holds as much aesthetic importance as its shape and outward projection. The Frankfort horizontal plane serves as an effective reference point to discern the ear's orientation in relation to facial features such as the eyebrows, eyes, and nose [13]. This plane intersects the lower orbital rim and extends to the topmost part of the tragus [13]. Along this horizontal plane, the upper limit of the helical rim aligns with the lateral brow, running parallel to the Frankfort line, whereas the earlobe is level with the tip of the nose. In terms of vertical positioning, the auricle leans about 15 to 30 degrees towards the back and side [11], granting the ear its slightly angled look when viewed laterally.

Ears that exceed these standard measurements can appear disproportionately large and stand out. While unilateral abnormalities are evident due to the resulting asymmetry, bilateral issues are noticeable when the ears diverge significantly from common dimensions. A deep-seated appreciation for the anatomy and relative positioning of the ear is crucial for its accurate evaluation and subsequent surgical intervention, as elaborated below.

Postauricular approach

The postauricular approach serves as a foundational technique in ear surgery, primarily due to its direct access to the labyrinth and infratemporal fossa. Several vital surgical interventions, such as mastoidectomy, tympanoplasty, endolymphatic sac decompression, cochlear implantation, vibrant soundbridge implantation, semicircular canal obliteration, and facial nerve decompression, can be accomplished through this approach [14-17]. Moreover, radical excisions for pathologies like endolymphatic sac carcinoma and the reparative procedures for cerebrospinal fluid otorrhea further emphasize its versatility [18].

The postauricular approach is initiated with a curving incision parallel to the retroauricular groove, starting from the superior border of the auricle's root to the mastoid tip. Key surface landmarks, including the temporal line, suprameatal spine, suprameatal triangle, cribriform area, parietal notch, and mastoid tip, are crucial for ensuring accurate and safe surgical progression [4]. These landmarks help orient the surgeon, with the temporal line indicating the bottom of the middle cranial fossa, the suprameatal triangle marking the mastoid antrum, and the line between the mastoid tip and parietal notch denoting the position of the sigmoid sinus [4]. For the successful execution of the postauricular approach, surgical finesse is coupled with a rigorous understanding of the anatomy. While the procedure itself is minimally invasive, surgeons should be careful to ensure that the integrity of the auricle's connective tissues, nerves, and blood vessels is preserved to prevent postoperative complications and maintain the ear's natural form and function.

Mechanisms leading to auricular protrusion postsurgery

Auricular protrusion after surgical interventions can be an undesirable complication. This anomaly may stem from various mechanisms, including scar contracture, inadequate cartilage reshaping, or postoperative edema. Scar contracture, which occurs when fibrous tissue tightens over time, can pull the ear forward, causing it to protrude. It was observed that excessive scarring might lead to distortion of the ear's natural shape [19]. Another crucial mechanism is inadequate reshaping or removal of cartilage. If the surgeon fails to shape the cartilage appropriately or if there's a lack of symmetry in the modifications, it can result in a prominent ear postsurgery. It was found that the meticulous technique during cartilage reshaping is vital to prevent postoperative protrusion [20]. Lastly, postoperative edema, or swelling, is a transient cause that can give the appearance of auricular protrusion until the swelling subsides [21]. Additionally, an underdefined antihelical fold during surgery results in the upper and middle parts of auricular protrusion. If the concha is especially deep, it primarily pushes the middle section of the auricle forward. In rarer cases, an exaggerated lobule can make the lower part of the ear appear more prominent. While these abnormalities can individually cause the ear to protrude, they often coexist, intensifying the overall protrusion of the auricle [22].

Factors contributing to increased risk

Certain factors amplify the risk of postoperative auricular protrusion. Firstly, the surgical technique and experience play a paramount role. Inexperienced surgeons or those not adhering to several techniques can inadvertently lead to poor outcomes. Calder and Naasan stressed the importance of selecting experienced surgeons to reduce the risk of complications. Patient factors such as age, skin elasticity, and individual healing capacities can also influence results. For instance, younger patients with more elastic skin may experience different healing patterns than older individuals [23]. An underlying genetic predisposition to scar heavily, known as keloid or hypertrophic scarring, can also contribute to pronounced auricular protrusion postsurgery. It was indicated that individuals with a history of keloid or hypertrophic scars might need to approach otoplasty with caution due to the risk of excessive postoperative scarring [24]. Lastly, postoperative care, including how the patient adheres to care instructions and how they sleep, can influence the outcome. As per Furnas, avoiding pressure on the ears postsurgery and following postoperative care guidelines can considerably reduce the risk of complications [25]. To minimize the risk of postoperative auricular protrusion, it is crucial to choose a qualified and experienced surgeon who is well-versed in otoplasty techniques. Patients should also have realistic expectations about the outcomes and engage in open communication with their surgeons regarding their desired results and concerns.

Auricular protrusion after the postauricular approach

There is very limited evidence regarding the incidence of auricular protrusion after the postauricular approach. Kim conducted a prospective study to evaluate the changes in auricular protrusion after chronic otitis media surgery with the postauricular approach [21]. He included 47 patients who underwent tympanoplasty (n=7), canal wall-up mastoidectomy (n=17), and canal wall-down mastoidectomy (n=23). The extent of the auricular protrusion was evaluated using a specific technique detailed in the Mashhadi study [26]. Initially, measurements were taken from the mastoid process to the outer edge of the helix, aligning with the plane at the top part of the tragus, termed the mid-auricle region (MD). Subsequently, the greatest distance from the head to the helical rim was recorded, referred to as the upper ear region (UP). A single individual (the author) took these measurements at distinct intervals: a day before surgery, on the day following surgery, two weeks postsurgery, and then at 1, 2, 4, and 6 months after the procedure [21]. Measurements taken a day prior to surgery for the MD and UP showed averages of 16.9±5.17 mm and 24.1±5.16 mm, respectively. Two weeks after the surgery, the MD and UP increased to reach 19.9±4.54 mm and 28.1±4.45 mm, respectively. Notably, while significant differences from baseline were observed in the first-month postsurgery (p<0.001), measurements from the second (MD: 17.2±4.40 mm, UP: 24.9±4.14 mm) to the sixth month (MD: 15.9±4.35 mm, UP: 23.5±4.09 mm) postoperatively closely resembled baseline values (p>0.05), indicating stabilization or return to pre-surgery states [21].

Surgical procedures were grouped into two: Group 1 included tympanoplasty and canal wall-up mastoidectomy, whereas Group 2 was reserved for canal wall-down mastoidectomy. For Group 1, noticeable variances in MD measurements were seen up to two months postsurgery compared to initial values, but by 4-6 months, these measures closely matched the baseline. Conversely, Group 2 displayed significant measurement deviations from baseline only up to one month after surgery, and from 2-6 months postsurgery, measurements aligned with initial values. Both groups had notable differences in UP measurements between baseline and the 1-month postoperative mark, yet 2-6 months postsurgery measurements were consistent with initial findings. Specifically, Group 2, comprising patients who had canal wall-down surgeries, saw a trend in MD and UP values returning closer to baseline between 4 and 6 months postoperation [21].

The postsurgical peak in auricular protrusion at two weeks can be attributed to various factors, including edema from the posterior auricular approach. Although it was hypothesized that the most prominent auricular protrusion would occur immediately postoperation, the data indicated a peak in the second week. This discrepancy may be due to the mastoid compression dressing suppressing auricular protrusion on the first postoperative day. The support structures behind the ear, like subcutaneous tissue, muscle, and bone, influence its postoperative protrusion. Tympanoplasty largely leaves these intact, whereas canal wall-up mastoidectomy removes part of the mastoid bone but spares most supporting structures. Conversely, the canal wall-down mastoidectomy extensively disrupts these supports. The early return to the baseline in Group 2 may be due to postsurgical depression in the postauricular area [21].

Akgül et al. conducted a study to evaluate whether preferring a postauricular approach during ear surgery causes auricular protrusion over time [27]. For all patients, the graft material of choice was the temporal muscle fascia. After the surgery, only the skin and the layer beneath it were stitched up. A mastoid pressure dressing was employed for a period ranging from 24 to 48 hours postsurgery. After this dressing was in place, daily replacements were not done. On the seventh day after the procedure, skin stitches were removed. Patients were prescribed oral antibiotics and pain relievers for the subsequent week [27]. To evaluate the extent of auricular protrusion, we adopted the method detailed by Hong et al., focusing on the distances from the mastoid's surface to specific points on the helical rim [28]. They took measurements from the mastoid skin to the topmost part of the helical rim and from the mastoid skin to the midpoint of the helical rim, both before the surgery and a year after. Contrary to the technique used by Hong et al., they did not consider the distance from the mastoid skin to the lobule due to concerns over potential inaccuracies caused by the lobule's flexibility. Preoperative, the measures from the posterior point were 15.03±2.86 mm, and it was 14.67±3.12 mm one year postoperatively (p=0.327). Regarding the mid-point, there was also no significant (p=0.073) difference between preoperative and postoperative (17.92±2.96 mm vs. 17.25±3.17). These findings indicated that the postauricular approach was not associated with postoperative auricular protrusion [27].

Hong et al. conducted a prospective study on patients who underwent tympanomastoidectomy or tympanoplasty using a postauricular approach. Ear position was evaluated, as described before, preoperatively and at postsurgery intervals of one day, three months, and a year. Depending on the case, tympanoplasty with canaloplasty and/or cortical mastoidectomy was performed, using either temporalis fascia or tragal cartilage as grafts. The closure occurred in layers, with a mastoid head dressing applied postsurgery and subsequently removed on the first postoperative day. Preoperatively, the measures at the superior most aspect of the helix were 10.95±1.31mm; at the midpoint, it was 14.95±1.08mm; at the lobule, it was 19.21±1.58mm. One year after the surgery, the measures were even lower than preoperative, with no significant difference at the three points (p>0.05) [28].

On the other hand, Okur et al. conducted a prospective randomized controlled trial on 46 patients who underwent tympanoplasty and tympanomastoidectomy with or without postoperative mastoid dressing [29]. They were divided into two groups: 17 patients (mean age 23.88 years) received a mastoid dressing that applied pressure using a circumferential bandage, while 20 patients (mean age 31.2 years) had a simple dressing without compression. Senior surgeons performed all procedures, and incisions were closed using a combination of absorbable and non-absorbable sutures. Postoperatively, all patients received oral antibiotics for two weeks, with dressings and sutures removed after one week. Three months postsurgery, using a plastic caliper compass, the distance from the mastoid scalp to the upper helix rim was measured on both operated and non-operated ears, ensuring the patient's head remained level during measurements [29]. Their findings showed that in the mastoid dressing group, eight patients had a tympanoplasty with mastoidectomy, eight had only a tympanoplasty, and one was solely a mastoidectomy. For the no-mastoid dressing group, seven underwent tympanoplasty with mastoidectomy, twelve had only a tympanoplasty, and one had just a mastoidectomy. Tragal cartilage was the graft of choice for six patients in the mastoid dressing group and 18 in the no-mastoid dressing group, while temporal fascia was preferred for 11 and one patient in the respective groups. Only one minor skin complication arose in the mastoid dressing group, with no complications in the no-mastoid dressing group. Analyses revealed no significant differences in the distance from mastoid to helix between or within the groups, regardless of whether mastoidectomy was performed [29].

Discussion

There is a longstanding belief that making a postauricular incision during ear surgeries could lead to auricular protrusion. However, the number of studies that investigated this point is very limited. Despite this limitation, almost all of these studies agreed that the postauricular approach is not associated with the occurrence of auricular protrusion. Even in the studies that reported some cases of protrusion, the auricle returned to its normal position within a month postoperatively. These findings suggest that surgeons should continue to use the postauricular approach without any concerns regarding auricular protrusion; however, it is recommended to take all precautionary measures, including appropriate patient selection, experienced surgeon, and postoperative dressing.

In the study of Akgül et al., they included patients who underwent tympanoplasty without mastoid surgery, which is quite different from the patients' selection in the studies that showed postoperative auricular protrusion [27]. When making a postauricular incision, the skin, subcutaneous layer, postauricular muscles, and mastoid periosteum are successively incised. It is believed that stitching these layers after the procedure might help in preventing auricular protrusion due to contraction. However, postoperative auricular protrusion is often observed, especially in those undergoing mastoidectomy. In Akgül's study, only the skin and the layer beneath it were stitched, leaving deeper structures like the postauricular muscle unstitched [27]. Another point of consideration with a postauricular incision is the necessity of a mastoid dressing postsurgery. Akgül et al. believe that mastoid dressing is critical in preventing postoperative auricular protrusion. In contrast, Okur et al. argued that such a dressing does not prevent auricular protrusion postoperatively. Although all their study participants had a pressure dressing for 24 to 48 hours postsurgery, its main objective was to ward off hematoma development on the temporal muscle rather than to prevent auricular protrusion [29]. According to Hong et al., the slight change in the auricle on the first day postoperatively is mainly attributed to the edema. The local swelling as a result of soft tissue dissection may place pressure on the compliant ear, forcing it into a more prominent position. As observed in several studies, the change in auricular position resolves after the disappearance of local edema.

In the normal ear, the angle between the ear and the head, or the auriculo-cephalic angle, ranges from 25° to 30°. In a study by Graham et al., the average distance between the ear's outer rim and the scalp was found to be 11 mm at the top of the helix and 13.5 mm at its midpoint [30]. In a local study, Okur et al. found this distance to be an average of 16.9 mm. Our findings showed an average distance of 15.03 mm from the mastoid to the top of the helix and 17.92 mm at the helix's midpoint [29]. Results obtained by Akgul et al. align with the study by Okur et al., suggesting that variations in measurements across studies may be due to differing physical characteristics of participants or measurement methods [27].

Clinical implications

Patient Selection and Individual Variation

The postoperative outcomes of auricular protrusion after otoplasty or tympanoplasty can be influenced by various factors, including the surgical technique, individual healing capacities, and underlying genetic predisposition. This highlights the importance of a comprehensive preoperative assessment, which includes understanding the patient's individual risk factors, such as predisposition to keloid or hypertrophic scarring. Surgeons need to provide comprehensive counseling regarding potential outcomes and set realistic postoperative expectations.

Surgical Techniques and Experience

Surgical techniques, particularly in cartilage reshaping and the choice of postauricular approach, play a significant role in the postoperative outcomes. The varying results and findings from the mentioned studies indicate that the surgeon's experience, surgical techniques, and postoperative care play paramount roles in the final outcome. Surgeons must be well-versed in the various techniques of otoplasty and tympanoplasty and must be meticulous in technique during cartilage reshaping. Additionally, considering that postoperative edema peaks in the second week, appropriate postsurgical dressing, especially the mastoid compression dressing, can be beneficial in mitigating undue protrusion.

Future directions

Standardization of Measurement Techniques

There are multiple techniques and points of measurement detailed by various studies to determine auricular protrusion. The lack of a standardized technique can lead to challenges in comparing and contrasting results across studies. Future studies should aim for standardization in the method of measuring auricular protrusion to achieve more cohesive data interpretation.

Long-Term Outcomes and Comparative Studies

While some studies have touched upon the long-term outcomes (one-year postsurgery), there is a need for longitudinal studies to truly understand if the postsurgical changes are permanent or if there are further variations down the line. Moreover, comparative studies examining various surgical techniques, approaches, and postsurgical care can provide a more comprehensive understanding of which methodologies yield the best outcomes. Such studies would be instrumental in guiding surgical decision-making and patient counseling.

Enhanced Postoperative Care

As the studies mentioned indicate the potential implications of postoperative care on outcomes; future research should also delve deeper into optimizing postoperative protocols. This includes studying the effects of varying durations of mastoid pressure dressings, the role of oral antibiotics, and other postoperative interventions that could potentially influence outcomes.

Innovative Surgical Techniques

With advancing medical technologies and the continuous evolution of surgical methodologies, it is essential for future research to evaluate and potentially develop novel techniques that can reduce the risk of complications and improve the overall results of ear surgeries.

## Conclusions

Our comprehensive review has thoroughly examined the intricacies surrounding the potential for auricular protrusion subsequent to the postauricular surgical approach, providing an in-depth exploration of the prevailing research available on this topic. The findings from multiple studies consistently indicate that while transient auricular protrusion may arise immediately postsurgery, it is neither persistent nor significant over the long term. This aligns with the broader surgical consensus, suggesting that the postauricular approach remains a safe and reliable method. However, its success hinges on various factors, emphasizing the significance of ensuring optimal surgical standards. It is vital for surgeons to evaluate each patient's unique physiological factors, such as age, skin elasticity, and predisposition to keloid or hypertrophic scarring. Recognizing these individual variances can guide surgical decisions and postoperative care plans, tailoring them to each patient's specific needs. The expertise of the operating surgeon is undeniably crucial. Ensuring that the surgical approach is conducted by well-trained, experienced professionals can drastically reduce the potential for complications. Furthermore, continuous training and updates on the latest techniques and findings can further elevate the standard of care provided. Adherence to stringent postoperative care, encompassing factors like avoiding undue pressure on the operated ear and following the surgeon's guidelines, is indispensable. Such adherence can significantly influence the outcome, preventing potential complications and promoting optimal healing. While the insights provided by our review are robust, the realm of medical science is ever-evolving. Continued investigation is paramount. As techniques evolve, tools are refined, and our understanding of the human body deepens, the approach to the postauricular method and its outcomes may evolve as well.
